# A Prospective Study Comparing Functional Imaging (^18^F-FDG PET) Versus Anatomical Imaging (Contrast Enhanced CT) in Dosimetric Planning for Non-small Cell Lung Cancer

**DOI:** 10.22038/aojnmb.2017.8706

**Published:** 2017

**Authors:** Archana Prathipati, Ranadheer Gupta Manthri, Bala Venkat Subramanian, Pranabandhu Das, Swapna Jilla, Sangeetha Mani, Anitha Kumari J., Settipalli Sarala, Radhika Kottu, Tek Chand Kalawat, Kotiyala Venkata Jagannath Rao Naidu

**Affiliations:** 1 Department of Radiation Oncology, Sri Venkateswara Institute of Medical Sciences, Tirupati, Andhra Pradesh, India; 2 Department of Nuclear Medicine, Sri Venkateswara Institute of Medical Sciences, Tirupati, Andhra Pradesh, India; 3 Department of Radio Diagnosis, Sri Venkateswara Institute of Medical Sciences, Tirupati, Andhra Pradesh, India; 4 Department of Pathology, Sri Venkateswara Institute of Medical Sciences, Tirupati, Andhra Pradesh, India

**Keywords:** ^18^F-fluorodeoxyglucose positron emission tomography, Lung cancer, Target volume delineation

## Abstract

**Objective(s):**

^18^F-fluorodeoxyglucose positron emission tomography/computed tomography (^18^F-FDG PET-CT) is a well-used and established technique for lung cancer staging. Radiation therapy requires accurate target volume delineation, which is difficult in most cases due to coexisting atelectasis. The present study was performed to compare the ^18^F-FDG PET-CT with contrast enhanced computed tomography (CECT) in target volume delineation and investigate their impacts on radiotherapy planning.

**Methods:**

Eighteen patients were subjected to ^18^F- FDG PET-CT and CECT in the same position. Subsequently, the target volumes were separately delineated on both image sets. In addition, the normal organ doses were compared and evaluated.

**Results:**

The comparison of the primary gross tumour volume (GTV) between the ^18^F-FDG PET-CT and CECT imaging revealed that 88.9% (16/18) of the patients had a quantitative change on the ^18^F-FDG PET-CT. Out of these patients, 77% (14/18) of the cases had a decrease in volume, while 11% (2/18) of them had an increase in volume on the ^18^F-FDG PET-CT. Additionally, 44.4% (8/18) of the patients showed a decrease by > 50 cm^^3^^ on the ^18^F-FDG PET-CT. The comparison of the GTV lymph node between the ^18^F-FDG PET-CT and CECT revealed that the volume changed in 89% (16/18) of the patients: it decreased and increased in 50% (9/18) and 39% (7/18) on the ^18^F-FDG PET-CT. New nodes were identified in 27% (5/18) of the patients on the ^18^F-FDG PET-CT. The decrease in the GTV lymph node on the ^18^F-FDG PET-CT was statistically significant. The decreased target volumes made radiotherapy planning easier with improved sparing of normal tissues.

**Conclusion:**

GTV may either increase or decrease with the ^18^F-FDG PET-CT, compared to the CECT. However, the ^18^F-FDG PET-CT-based contouring facilitates the accurate delineation of tumour volumes, especially at margins, and detection of new lymph node volumes. The non-FDG avid nodes can be omitted to avoid elective nodal irradiation, which can spare the organs at risk and improve accurate staging and treatment.

## Introduction

According to the Indian Council of Medical Research population-based cancer registries, 10-12% of cancer patients have lung cancer, which is the most common cancer among males (1). Based on the hospital-based cancer registries, the lung cancer is one of the top three and top ten cancers among males and females, respectively (2). Surgery is the treatment of choice for medically fit patients with early-stage lung cancer. Nevertheless, medically inoperable stages I, II, and III lung cancers are primarily treated by radiotherapy (RT) either with or without chemotherapy (3). In many studies, five-year survival for patients with stage I disease exceeded 60%; however, in stage III disease, it varied around 15% (4, 5).

The locoregional control of lung cancer, achieved either by surgery and/or chemoradiotherapy, plays an important role in the treatment of this cancer, which in turn may contribute to better survival. Therefore, it is frequently emphasized that better RT techniques are needed to provide improved targeting of the tumour and optimally spare the adjacent normal tissues and uninvolved lung. However, it is essential to accurately identify tumour volumes in order to have improved RT techniques. The goal is to prevent missing marginal tumour tissue, which highlights the importance of better imaging for volume delineation.

Traditionally, the target volumes are delineated on computed tomography (CT) images with fusion of magnetic resonance imaging (MRI) when available. Nevertheless, the lung MRI is not used due to artefacts caused by respiratory motion. Lung tumours, especially squamous cell tumours, are more commonly located centrally and cause atelectasis, which is difficult to differentiate from the tumour itself on the planning CT images. This leads to large target volumes with increased radiation toxicity. Additionally, relatively large mediastinal nodes may not actually harbour metastasis, and if they are included in the target volume, it may lead to increased toxicity. Therefore, functional imaging such as ^18^Fluorine-labelled fluorodeoxyglucose positron emission tomography co-registered with computed tomography (^18^F-FDG PET-CT) may facilitate the delineation (4). Apart from volume delineation, the ^18^F-FDG PET-CT is proven to accurately stage the disease and improve the treatment decision making.

Therefore, this study was undertaken in order to investigate the impact of functional imaging on target volume delineation and RT planning. To this aim, we compared the functional imaging (i.e., ^18^F-FDG PET-CT) with anatomical imaging (i.e., contrast enhanced computed tomography [CECT]) in the gross tumour volume (GTV) delineation of non-small cell lung cancer (NSCLC). Additionally, dosimetric measurements were taken of the adjacent organs at risk (OAR) in the RT treatment planning of NSCLC.

## Methods

This prospective study was conducted on 40 NSCLC patients during April 2014-July 2015 in a tertiary care hospital after obtaining approval from the Institutional Ethics Committee. The NSCLC patients, diagnosed via histopathology, were subjected to ^18^F-FDG PET-CT in the treatment position, i.e., supine with hands above the head with three reproducible fiducial markers. If proven to be non-metastatic (18 patients), a planning CECT was performed in the treatment position.

The ^18^F-FDG PET-CT and CT imaging (3-mm slices) were implemented during quiet breathing from the base of the skull through the proximal thighs at 110 kVp and 70-110 mA. The PET images were obtained over the same anatomic extent beginning 45-60 min after the administration of 0.2 mCi/kg bodyweight (BW) of ^18^F-FDG. Accordingly, 8-10 bed positions were imaged per patient, depending on the patient height with imaging times of 2-4 min per bed position.

Subsequently, within one week, a planning CECT (Somatom Definition AS 16-slice multidetector, Siemens, Germany) was performed in the treatment position with intravenous administration of non-ionic contrast at 1-2 mL/kg of BW. The CT images (3-mm slices) were typically obtained during quiet breathing from the chin to the umbilicus at 110 kVp and 70-100 mA.

The ^18^F-FDG PET-CT and planning CECT images were imported to the Eclipse treatment planning system version 2007. Then, the target volumes were contoured according to the international commission on radiological units and measurements (ICRU) report 50. Contouring was performed on the CECT images without utilising the ^18^F-FDG PET-CT images to avoid bias.

### Contouring on CECT

The GTV contouring was performed for the primary tumour and lymph nodes. The primary tumour was outlined using both lung window settings (W=1,600, C=600) and mediastinal window settings (W=400, C=40) to optimise the definition at the interfaces with normal structures. Bone windows were used if the tumour abutted or involved the bone (W=1,600, C=400). Furthermore, the lymph nodes were defined using the mediastinal windows. An involved lymph node was defined as having a size of more than 1 cm on the short-axis diameter. The CECT-GTV encompassed the GTV of primary tumour and lymph node. The clinical target volume (CTV) was contoured on the CECT by a uniform outgrowth of the CECT-GTV by 1.5 cm, and the margin was reduced near critical structures like the spinal cord. The planning target volume (PTV) on the CECT was created by an isotropic outgrowth of a 5 mm margin circumferentially.

### Contouring on ^18^F-FDG PET-CT

The target volumes were contoured on the CT component of the ^18^F-FDG PET-CT with the help of PET. The treating radiation oncologist and nuclear medicine physician determined the tumour delineation based on the ^18^F-FDG PET-CT via visual interpretation. The window settings were adjusted with view parameter at hot iron mode, and mass with greater activity than mediastinal blood pool activity was contoured.

For the patients with a collapsed lung, the presence of ^18^F-FDG-avid disease on the planning ^18^F-FDG PET-CT scan was used to define an anatomic margin. The lymph nodes with ^18^F-FDG uptake on the ^18^F-FDG PET-CT scan were considered as positive.

The distinction between the benign-appearing lymph nodes versus malignant-appearing lymph nodes was based on a greater intensity of ^18^F-FDG PET-CT uptake, compared to the mediastinal blood pool.

The PET-CT-based GTV encompassed the PET-CT-based GTV of the primary tumour and lymph nodes. The PET-CT-based CTV and PET-CT-based PTV were contoured in a similar way to the CECT-based CTV and CECT-based PTV. The delineated OAR were as follows:

Lungs: automatically delineated on the Eclipse treatment planning workstation, and then manually modified to exclude the trachea and bronchi;

Heart: delineated from the bottom of the aortic arch to the bottom of the heart;

Spinal cord: delineated slice by slice after adjusting the CT window width and level to clearly demonstrate the spinal cord;

Oesophagus: delineated from the level of the cricoid cartilage to the area above the oesophagogastric junction.

The target volumes were planned with three-dimensional conformal radiotherapy (3D-CRT). Accordingly, PTV received 95% of the target dose, and normal organ doses fell within the tolerance limits recommended by the Quantitative Analysis Of Normal Tissue Effects In The Clinic (QUANTEC). The study parameters, namely GTV (i.e., GTV of primary tumour and lymph nodes), mean lung dose (MLD), lung volume receiving more than 20 gray (V20), mean oesophageal dose (MED), mean heart dose (MHD), heart V30, and maximum spinal cord dose (MSD) were compared on CECT and ^18^F-FDG PET-CT. The median values were derived and analyzed using the Mann-Whitney U test, which is a non-parametric statistical test, through the SPSS software version 20. The P-value less than 0.05 was considered statistically significant.

## Results

Forty patients with histopathologically proven NSCLC during the study period were screened for inclusion into the study. Out of these patients, 14 cases were excluded from the study for several reasons including obvious metastatic disease, renal failure, and contrast allergy. After the exclusion, 26 patients were subjected to ^18^F-FDG PET-CT in the treatment position. Eight patients were diagnosed with metastatic disease on the ^18^F-FDG PET-CT; as a result, the treatment intent was changed from curative to palliative, and they were excluded. Finally, 18 patients were recruited into the study. These patients were subjected to planning CECT in the same position, and volumes were separately contoured and planned.

### Stage migration

When the individual T, N, and M stages of each subject on the ^18^F-FDG PET-CT imaging were compared to the CECT imaging, the following changes were identified on the ^18^F-FDG PET-CT:

Tumour status (T) was downstaged in 2/18 (11%) patients.

Lymph node status (N) was upstaged in 7/18 (39%) patients.

Lymph node status (N) was downstaged in 3/18 (17%) patients.

Metastatic status (M) (contralateral lung nodules) was downstaged in 4/18 (22%) patients.

Out of the 18 participants, 17 were male. The age of presentation varied from 50-70 years with a mean age of 60.33 years. Pulmonary involvement was predominantly right-sided with a right to left ratio of 2:1. Stage-wise distribution showed that the majority of the patients (56%, 10/18) presented stage IIIA. Furthermore, site-wise distribution showed that most of the patients (62%, 11/18) had tumour in the upper lobe. Histopathologically, the majority of the patients (62%, 11/18) had an adenocarcinoma located peripherally in the lung.

### Comparison of the GTV of primary tumour volume on the ^18^F-FDG PET-CT and CECT (Table 1)

**Table 1:** GTV changes in the present study

Volume	Changed^#^	Increased^#^	Decreased^#^
GTV primary tumor	88.8% (16/18)	11.1%(2/18)	77.7%(14/18)
GTV lymph nodes	88.8%(16/18)	38.8%(7/18)	50%(9/18)

# Number of subjects showing volume change out of number of subjects studied

**Figure 1:**
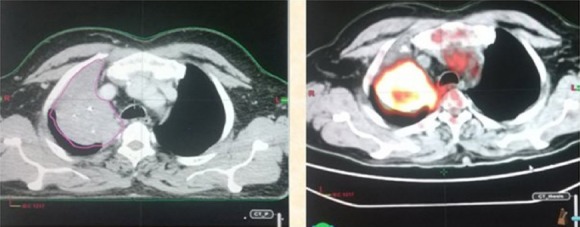
GTV primary tumor volume drawn in magenta on CECT and red on ^18^F-FDG PET-CT showing decrease in GTV volume on ^18^F-FDG PET-CT

**Figure 2:**
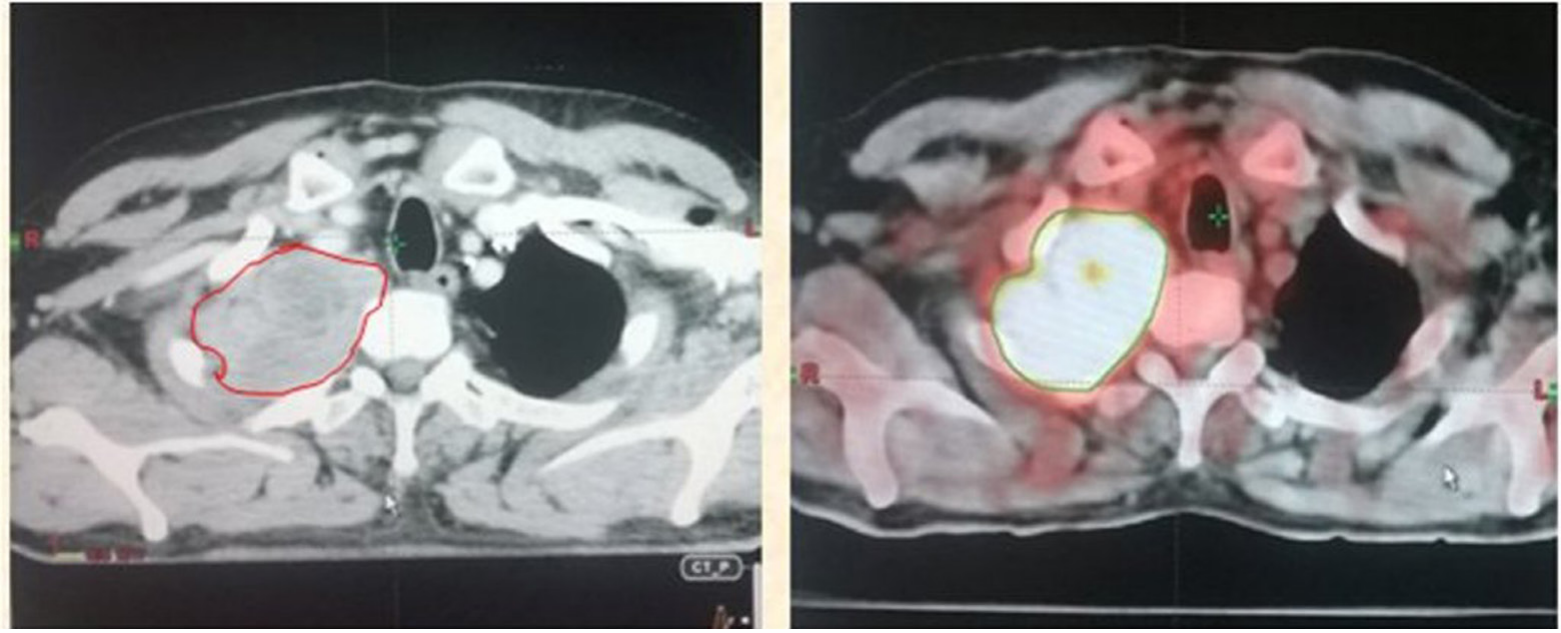
GTV primary tumor volume drawn in red on CECT and green on ^18^F-FDG PET-CT showing increase in GTV volume on ^18^F-FDG PET-CT

**Figure 3:**
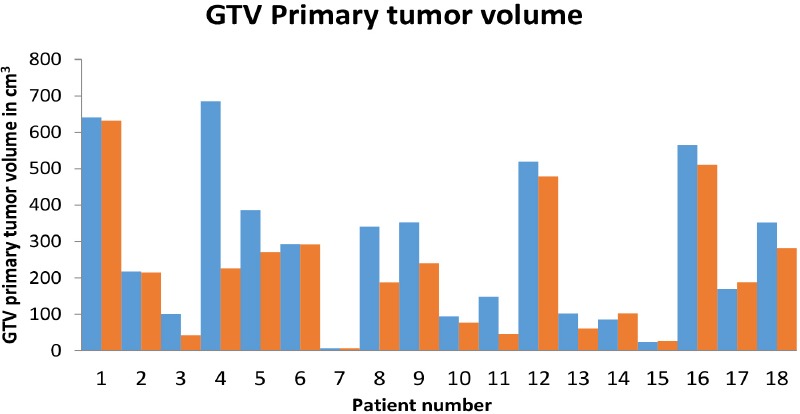
Bar chart with X –axis showing patient number and Y- axis showing GTV primary tumor volume in Cm^3^, GTV CECT primary tumor volume in blue and GTV PET CT primary tumor volume in red

Out of the 18 patients, 16 cases showed a quantitative tumor volume change on the ^18^F-FDG PET-CT, nevertheless, no volume change was observed in two patients. In addition, 77% (14/18) of the participants showed a decrease in the GTV on the ^18^F-FDG PET-CT. Out of these subjects, 44% (8/18) of the cases had a reduction by > 50 cm^3^ on the ^18^F-FDG PET-CT, compared to the CECT (Figure 1). In addition, the GTV of primary tumor increased on the ^18^F-FDG PET-CT in 11% (2/18) of the participants (Figure 2, 3).

### Comparison of GTV lymph node on the ^18^F-FDG PET-CT and CECT (Table 1)

**Figure 4:**
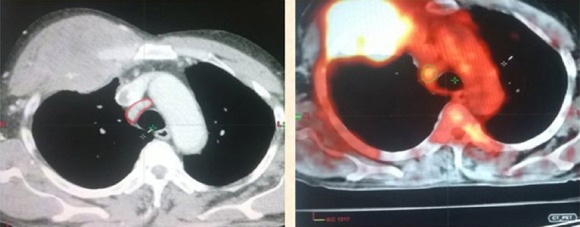
GTV lymph node volume drawn in red on CECT and green on ^18^F-FDG PET-CT showing decrease in GTV volume on ^18^F-FDG PET-CT

**Figure 5:**
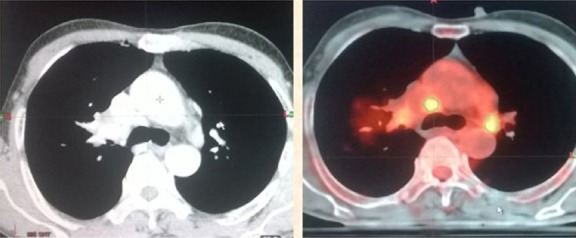
GTV lymph node volume drawn in red on CECT and green on ^18^F-FDG PET-CT showing increase in GTV volume on^18^F-FDG PET-CT

**Figure 6:**
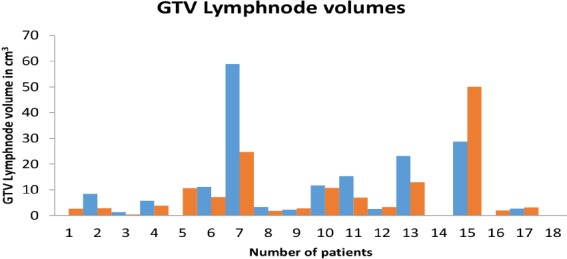
Bar chart with X –axis showing patient number and Y- axis showing GTV lymph node volume in cm^3^, GTV CECT lymph node volume in blue and GTV PET CT lymph node volume in red

The GTV lymph node changed in 89% (16/18) of the patients on the ^18^F-FDG PET-CT imaging. Out of these patients, 50% (9/18) and 39% (7/18) of the participants showed decrease (Figure 4, 5) and increase (Figure 6) in the GTV lymph node, respectively. Furthermore, new nodes were identified in 27% (5/18) of the patients on the ^18^F-FDG PET-CT imaging. However, two patients had no lymph node involvement on any imaging.

### Impact on planning

Although there was a volume change on the ^18^F-FDG PET-CT, the same RT plan, generated on the CT-based volumes, can have adequate coverage when used on the ^18^F-FDG PET-CT-based volumes. However, if the coverage is suboptimal on the ^18^F-FDG PET-CT, a new plan is generated. Accordingly, in the present study, we applied the same plan when dose coverage was suboptimal in 50% (9/18) of the patients and, replanning was required.

In the present study, all OAR doses were within the tolerance limits. When the OAR impact was analysed, all the patients had an increase in such parameters as MLD, V20, MHD, V30, and MED as well as a decrease in the MSD on the ^18^F-FDG PET-CT-based plan (Table 2, 3). However, the analysis of the data related to the patients with decreased GTV of primary tumor on the ^18^F-FDG PET-CT revealed an increase in the OAR parameters including MLD, V20, MHD, V30, and MED and a decrease in the MSD due to the increase in the GTV lymph node volume (Table 4, 5). Nevertheless, all OAR doses were within the normal limits.

**Table 2:** Target volume data in all subjects

Parameter^#^	CECT^$^	^18^F-FDG PET-CT^$^	P-value
GTV primary tumor	254.78	201.42	0.296
GTV lymph nodes	3.0	3.22	0.824

# Volume in cm^3^

$ Median value

**Table 3:** Dosimetric parameters for OAR in all subjects

Parameter	CECT^$^	^18^F-FDGPET-CT^$^	P-value
MLD^#^	1177.9	1339.9	0.343
MHD ^#^	780.1	1258.2	0.448
MED ^#^	1674.95	2605.05	0.411
Maximum spinal cord dose ^#^	4629.65	4387.25	0.776
V 20 of Lung^*^	18.85	21.03	0.189
V 30 of Heart^*^	8.0	19.37	0.251

# Dose in centi Gray (one Gray is 100 centi Gray)

* Percentage of volume

$ Median value

**Table 4:** Target volume data in subjects with decreased GTV primary tumor volume on ^18^F-FDG PET-CT (14/18)

Parameter^#^	CECT^$^	^18^F-FDG PET CT^$^	P-value
GTV primary tumor	346.49	232.85	0.150
GTV lymph nodes	2.94	3.54	0.769

# Volume in cm^3^

$ Median value

**Table 5:** Dosimetric parameters for OAR in subjects with decreased GTV primary tumor volume on ^18^F-FDG PET-CT

Parameter	CECT^$^	^18^F-FDGPET-CT^$^	P-value
MLD^#^	1248.1	1387	0.352
MHD^#^	1264.5	1580.75	0.401
MED^#^	1948.1	3098.65	0.265
Maximum spinal cord dose^#^	4566.45	3648.45	0.667
V 20 of lung^*^	18.85	25.49	0.194
V 30 of heart^*^	15	21.10	0.210

# Dose in centi Gray (one Gray is 100 centi Gray)

* Percentage of volume

$ Median value

In the present study, the analysis of the OAR impact among the patients with decreased GTV lymph node on the ^18^F-FDG PET-CT indicated an increase in such OAR parameters as MHD and heart V30. Furthermore, this analysis showed a decrease in parameters like MLD, lung V20, MED, and MSD (Table 6, 7). However, there was a decrease in the GTV of primary tumor due to the presence of a bulky disease in the lungs. Consequently, the plan was optimized towards sparing the lung given the history of chronic pulmonary disease in these patients.

**Table 6:** Target volumes in subjects with decreased GTV lymph node volume on ^18^F -FDG PET-CT (9/18)

Parameter	CECT	^18^F-FDG PET-CT	P-value
GTV lymph nodes	11.12	7	0.03673^@^
GTV primary tumor	148.53	77.18	0.222

@ P value of decrease in GTV lymph node volume on 18F FDG PET CT is statistically significant.

**Table 7:** Dosimetric parameters for OAR in subjects with decreased GTV lymph node volume on ^18^F-FDG PET-CT

Parameter	CECT^$^	^18^F-FDG PET-CT^$^	P-value
MLD^#^	1379.8	1350	1.000
MHD^#^	1196	1438.3	0.340
MED^#^	1784.3	1409	0.931
Maximum spinal cord dose^#^	4324.4	2831.3	0.863
V 20 of lung^*^	19.70	19.56	0.796
V 30 of heart^*^	13	21	0.297

# Dose in centi Gray (one Gray is 100 centi Gray)

* Percentage of volume

$ Median value

On the other hand, the analysis of the OAR impact among the patients with increased GTV lymph node on the ^18^F-FDG PET-CT revealed an increase in the OAR parameters like MLD, V20, MHD, V30, MED, and MSD (Table 8, 9). However, there was a decrease in the GTV of primary tumor on the ^18^F-FDG PET-CT due to an increase in the lymph node volume leading to a greater mediastinal area for treatment.

**Table 8:** Target volumes in subjects with increased GTV lymph node volume on ^18^F- FDG PET-CT (7/18)

Parameter	CECT	^18^F-FDG PET-CT	P-value
GTV primary tumor	385.45	290.97	0.710
GTV lymph nodes	2.31	3.15	0.073

**Table 9:** Dosimetric parameters for OAR in subjects with increased GTV lymph node volume on ^18^F-FDG PET-CT

Parameter	CECT^$^	^18^F-FDG PE -CT^$^	P-value
MLD^#^	1239.4	1595.9	0.142
MHD ^#^	326.3	488.34	0.535
MED^#^	2439.1	3421.1	0.209
Maximum spinal cord dose^#^	4804.1	4909.5	0.902
V 20 of lung ^*^	21.77	26.80	0.128
V 30 of heart^*^	0.2	6.71	0.314

# Dose in centi Gray (one Gray is 100 centi Gray)

* Percentage of volume

$ Median value

## Discussion

Around 10-15% of the newly registered patients had carcinoma lung in the hospital under investigation. The primary treatment of the early stage lung cancer is either surgery or stereotactic body radiotherapy. However, for the locally advanced disease, the RT with concurrent chemotherapy is the treatment of choice. Local control is necessary for a long-term disease-free survival. There are some measures to improve the local control including optimum tumour coverage with radiation by improving tumour delineation, improving RT planning technique, and precise delivery of radiation to the tumour (4).

In the 3D-CRT planning, the GTV delineation should be accurate because if contour is underdrawn or overdrawn, it may lead to local recurrence or increased normal organ doses, respectively. The main drawback during tumour delineation is the identification of atelectasis and loculated pleural effusion at the tumour-normal lung interface. The addition of functional imaging like ^18^F-FDG PET-CT clarifies tumour edges and simplifies delineation. If the target volume is relatively small and accurate, planning will be easy; therefore, the OAR will be spared, and even the dose escalation can be tried.

In our institute, we treat the locally advanced NSCLC patients with definitive radiation by 3D-CRT either with or without concurrent chemotherapy. We delineate target volumes based on the guidelines of the ICRU reports 50 and 62 on CECT imaging. Consequently, we compared the ^18^F-FDG PET-CT-based target volumes with the CECT-based target volumes and their impacts on the OAR.

### Impact on target volume delineation

In the 3D-CRT, the tumour volumes are delineated on the CT images. When the NSCLC patients have atelectasis or obstructive pneumonia, it is difficult to distinguish the boundaries between the incompletely expanded lung tissue and tumour tissue by conventional CT, which often results in inaccurate target delineation. Therefore, it leads to insufficient dose coverage of the target volume or too much damage to normal tissue.

According to the Radiation Therapy Oncology Group, the role of the elective lymph node irradiation is still an unresolved issue, and only involved nodes should be treated (5). The ^18^F-FDG PET-CT can effectively identify the boundary between the atelectasis and lung cancer, making the radiation target area precise. Therefore, it improves the local control, avoids unnecessary radiation injury, and reduces the radiation complications (6).

Toloza et al. conducted a pooled analysis over the sensitivities and specificities of the CT and PET, compared to the pathological staging of the mediastinum. For the CT, the pooled sensitivity and specificity were 0.57 and 0.82, respectively. For the PET, the pooled sensitivity and specificity were 0.84 and 0.89, respectively (7). Therefore, to improve the target volume delineation, especially of the mediastinum, we used the ^18^F-FDG PET-CT. In the present study, the ^18^F-FDG PET-CT-based contours showed both increase and decrease in the volume, compared to the CECT.

### Reason of the reduction in the GTV of primary tumour on ^18^F-FDG PET-CT

As shown in Figure 1, due to the associated collapse of lung tissue distal to the bronchial obstruction, the tumour is not differentiated from the atelectasis, which resulted in increased volume. However, as shown in the ^18^F-FDG PET-CT image, the ^18^F-FDG avidity facilitated the accurate identification of tumour margins by differentiating the tumour from atelectasis and decreasing the GTV of primary tumour volume on the ^18^F-FDG PET-CT-based contours.

### Reason of the increase in the GTV of primary tumour on ^18^F-FDG PET-CT

As illustrated in Figure 2, there is a high risk of marginal tumour miss due to the inability to accurately identify tumour margins near the chest wall and mediastinum. The ^18^F-FDG PET-CT imaging allowed for accurate identification of the mediastinum and chest wall involvement, which facilitated accurate delineation; however, it increased the tumour volume on the ^18^F-FDG PET-CT-based contours.

### Reason of the reduction in the GTV lymph node on ^18^F-FDG PET-CT

As presented in Figure 3, the mediastinal node greater than 1 cm in size on cross-section was considered involved and included in the GTV lymph node. However, the high negative predictive value of the ^18^F-FDG PET-CT decreased the GTV lymph node through accurate identification of the uninvolved nodes, therefore, it avoided the elective lymph node irradiation.

### Reason of the increase in the GTV lymph node on ^18^F-FDG PET-CT

As shown in Figure 4, the mediastinal node greater than 1 cm in size on cross-section was considered involved and included in the GTV lymph node volume. Nodes with central necrosis were also included in the target volume even if they were smaller than 1 cm. While it is difficult to identify small and indistinct nodes, the ^18^F-FDG PET-CT helps to detect the involved nodes, and therefore leads to an increase in the volume.

Bradley et al. studied 34 patients and found that the GTV lymph node was changed in 50% of the patients based on the ^18^F-FDG PET-CT. Furthermore, they showed that the risk of elective nodal failures was low, supporting the treatment of the involved fields (5). In a study conducted by Vanuytsel et al., it was shown that 62% of the patients (45/73 patients) had a change of treatment volume due to the employment of ^18^F-FDG PET-CT (8, 9). In addition, Deniaud-alexandre et al. studied the data of 92 patients and found that using ^18^F-FDG PET-CT data in RT planning led to a change of GTV in 50% of the patients with an increase and a decrease in 26% and 23% of them, respectively (10). Additionally, Hicks et al. studied 153 patients and documented GTV changes in 25% of the patients (11).

In the present study of 18 patients, there was a GTV of primary tumour change on the ^18^F-FDG PET-CT in 88.8% of the patients and a GTV of lymph node change on ^18^F-FDG PET-CT in 89% of the patients, which was higher than expected due to the small sample size. The decrease in the GTV lymph node on the PET-CT was statistically significant.

Applying the same RT plan of CECT on the ^18^F-FDG PET-CT dose coverage in the present study was suboptimal in 50% of the patients and needed replanning. Nestle et al. applied the same plan for PET-CT-based volumes to 34 patients and documented a change of plan for optimum coverage in 12 patients (35%) (12). Likewise, Kiffer et al. applied the same plan for PET-CT-based volumes to 15 patients and documented a change of plan for optimum coverage in four patients (26.7%) (13). In addition, Vanuytsel et al. applied the same plan on PET-CT-based volumes to 73 patients and documented a change of plan for optimum coverage in 45 patients (62%) (9). The present study demonstrated a change of plan in nine patients (50%) which is in agreement with the literature mentioned above.

### Impact on OAR

With the detection of new lymph nodes in the mediastinum, doses to the oesophagus, heart, and spinal cord will be expected to increase. Similarly, with a decrease in the tumour volume, normal organ doses are expected to decrease.

Bradley et al. found that RT planning based on the ^18^F-FDG PET-CT-based volumes decreased parameters like MLD (5). Additionally, Vanderwel et al. revealed that RT planning based on the ^18^F- FDG PET-CT-based volumes increased parameters of OAR doses like MHD and decreased parameters like lung V20, MLD, and MED (14). Yin et al. indicated that RT planning based on the ^18^F-FDG PET-CT-based volumes increased parameters of OAR doses including MED, MHD, and V30 in patients with increased GTV nodes. Furthermore, they reported that this planning decreased parameters like MLD, V20, and the maximum spinal cord dose (15). Deniaud-alexandre et al. found that RT planning based on the ^18^F-FDG PET-CT-based volumes decreased MSD, and the remaining OAR doses depended on the volume change (10).

In the present study, all OAR doses were within the tolerance limits; however, they showed some variations in accordance to the target volumes. Accordingly, the increase in the OAR dose led to the increase in the GTV of primary tumor. Furthermore, the decrease in the OAR dose resulted in a decrease in the GTV of primary tumor. It was also indicated that the increase and decrease in the OAR dose led to the increase and decrease in the GTV lymph node as explained previously.

## Conclusion

The PET-CT-based GTV may decrease or increase, compared to the CECT-based GTV. PET^-^CT-based contouring more accurately delineates tumour volumes (obstructive pneumonia and atelectasis), decreases contouring variability through more accurate tumour margins identification, and detects new lymph node volumes. Nodes, which are non-^18^F-FDG avid on the PET-CT could be omitted to avoid elective nodal irradiation, which can spare adjacent OAR. It also facilitates the accurate staging and treatment.

However, definite statistical statements cannot be made due to the small sample size and dosimetric nature of this study. Further randomised trials with larger sample size and a longer duration of follow-up are required to reach a consensus.
